# Soft Cerclage in Addition to Rigid Fixation for Surgical Management of Displaced Comminuted Midshaft Clavicle Fractures

**DOI:** 10.1002/atn2.70189

**Published:** 2026-07-31

**Authors:** Donovan A. Bronstein, Olivia M. Jochl, Jack H. Kramer, Eddie K. Afeste, Joseph J. Ruzbarsky

**Affiliations:** ^1^ The Steadman Clinic Vail Colorado U.S.A.; ^2^ University of North Carolina School of Medicine Chapel Hill North Carolina U.S.A.; ^3^ University of Illinois College of Medicine Rockford Illinois U.S.A.; ^4^ Wake Forest School of Medicine Winston Salem North Carolina U.S.A.

## Abstract

Midshaft clavicle fractures are one of the most common upper extremity fractures among adults. When displaced, open reduction and internal fixation may be indicated, most commonly performed using single or dual plating. Cerclage techniques can be used in addition to lag screws and plate fixation to better capture and reduce inferior and posterior fracture fragments that are typically difficult to capture using a lag screw technique. Although soft cerclage increases operative time and can pose a risk to neurovascular structures, better reduction of posterior and inferior fragments may promote healing and decrease nonunion rates.

## Graphical abstract

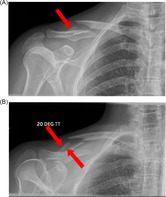

**VIDEO 1:** Technique for soft cerclage in addition to plate‐and‐lag screw fixation. The procedure begins with the patient in the beach chair position with their ipsilateral arm resting on their abdomen. After sterile preparation, an incision is made over the fracture site and dissected down to visualize the fracture. Point to point clamps are used for initial reduction of the fracture into anatomic alignment, followed by a lag screw and Synthes 2.7 mm plate fixation to stabilize the fracture. Ethibond #5 suture is passed around the clavicle inferiorly from anterior to posterior and caught using a using a curved hemostat. The suture is tensioned and then tied down to promote alignment of posterior and inferior butterfly fragments that could not be reduced from the lag screw or plate. This procedure is followed by copious irrigation, application of vancomycin, and layered closure. The patient is provided a sling postoperatively for comfort and typically has no range of motion restrictions. Video content can be viewed at https://doi.org/10.1002/atn2.70189.

The clavicle is the most commonly fractured upper extremity bone, accounting for 2.6% to 4% of all adult fractures, with more than two thirds of those at the midshaft.^1^ However, complications including nonunion and malunion occur at a rate of 4.8% in patients with displaced midshaft clavicle fractures,^2^ causing potential for pain and loss of shoulder function.^3^ In the case of displaced, comminuted midshaft clavicle fractures, open reduction and internal fixation is commonly used to promote union and increase early mobilization.^1^, ^2^, ^4^

Cerclage has been successfully implemented as secondary fixation in numerous long bones, including the humerus^5^ and femur^6^ and has been shown not to interfere with fracture healing.^7^ In this technical note and associated video, the authors describe their preferred technique for soft cerclage in addition to plate‐and‐screw fixation for the treatment of displaced, comminuted midshaft clavicle fractures.

## SURGICAL TECHNIQUE

### Indications and Preoperative Planning

**FIGURE 1:**
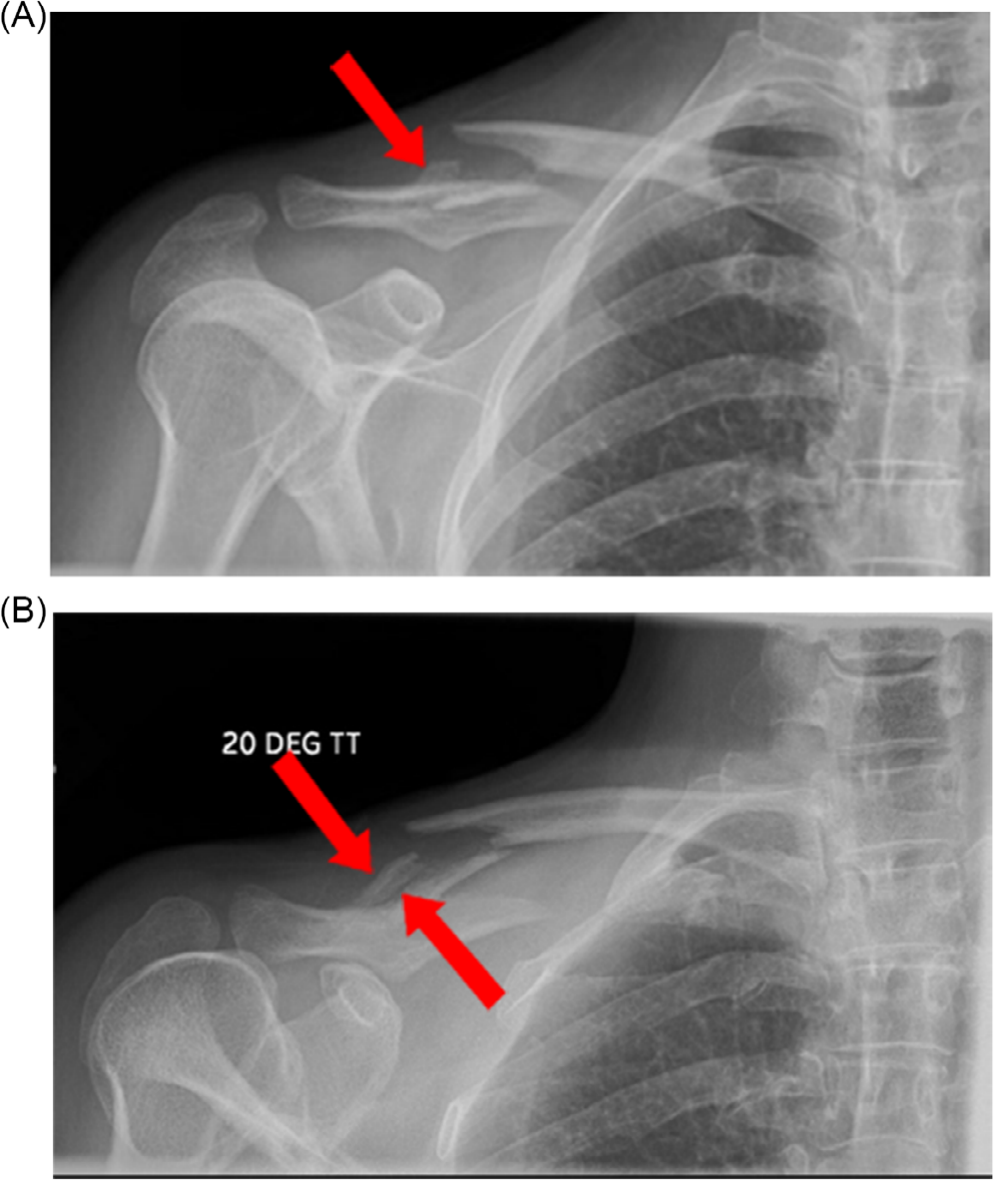
(A) Anteroposterior (AP) radiograph of the right clavicle with no tilt. (B) AP radiograph of the right clavicle with 20° of cephalad tilt. Red arrows are highlighting posterior butterfly fragments.

Indications for cerclage in the setting of a displaced comminuted midshaft clavicle fracture open reduction and internal fixation include posterior and/or inferior fragments that cannot be captured by lag screws or plates. Standard preoperative imaging includes anteroposterior clavicle radiographs with no tilt and 15° to 20° cephalad (Figure 1). Computed tomography scans may be used to better characterize displacement and orientation of fracture fragments.

### Preoperative Anesthesia

Patients undergo general anesthesia with the option of regional anesthesia for intraoperative and postoperative pain management. Patients may choose between an interscalene block using liposomal bupivacaine with or without a superficial cervical plexus block or local subcutaneous anesthesia comprised of a 1:1 mix of liposomal bupivacaine and 0.25% bupivacaine.

### Patient Positioning, Skin Preparation, and Surgical Draping

**FIGURE 2:**
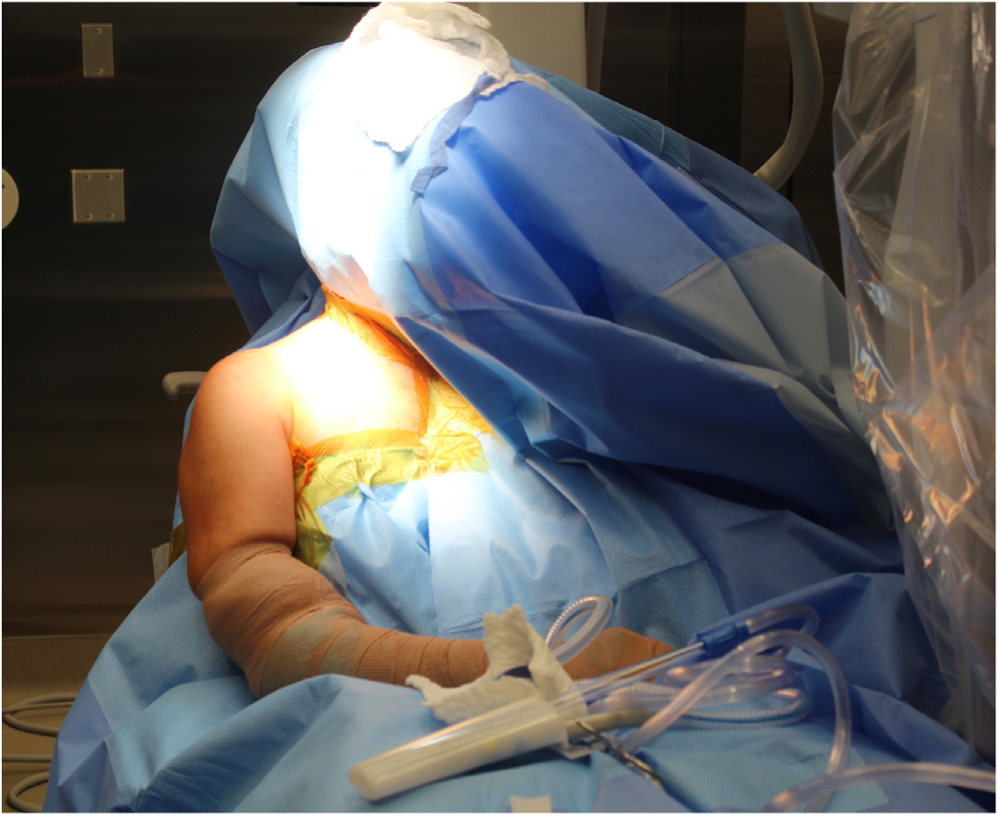
Patient is in the beach chair position with the entirety of the right clavicle exposed in the surgical field. A C‐arm (shown draped in the right‐hand side of the figure) is positioned on the contralateral side for ease of intraoperative fluoroscopy. The ipsilateral forearm is sterilely prepped and then rests on the abdomen.

Once anesthetized, the patient is placed in the beach chair position.^8^ A C‐arm is positioned over the contralateral side of the patient with the option to slide ipsilaterally to facilitate intraoperative fluoroscopy (Figure 2, Video 1). Chlorhexidine skin preparation is performed, followed by wide sterile draping, leaving both the distal and proximal ends of the clavicle in the surgical field. The ipsilateral arm is placed on the patient's abdomen.

### Incision and Dissection

A superficial incision is made over the midshaft clavicle fracture, followed by an incision of the platysma fascia and muscle. Care should be taken to ensure that any apparent branches of the suprascapular nerve are identified and protected. The incision is then carried down through the clavipectoral fascia to the fracture site.

### Fixation and Cerclage

**FIGURE 3:**
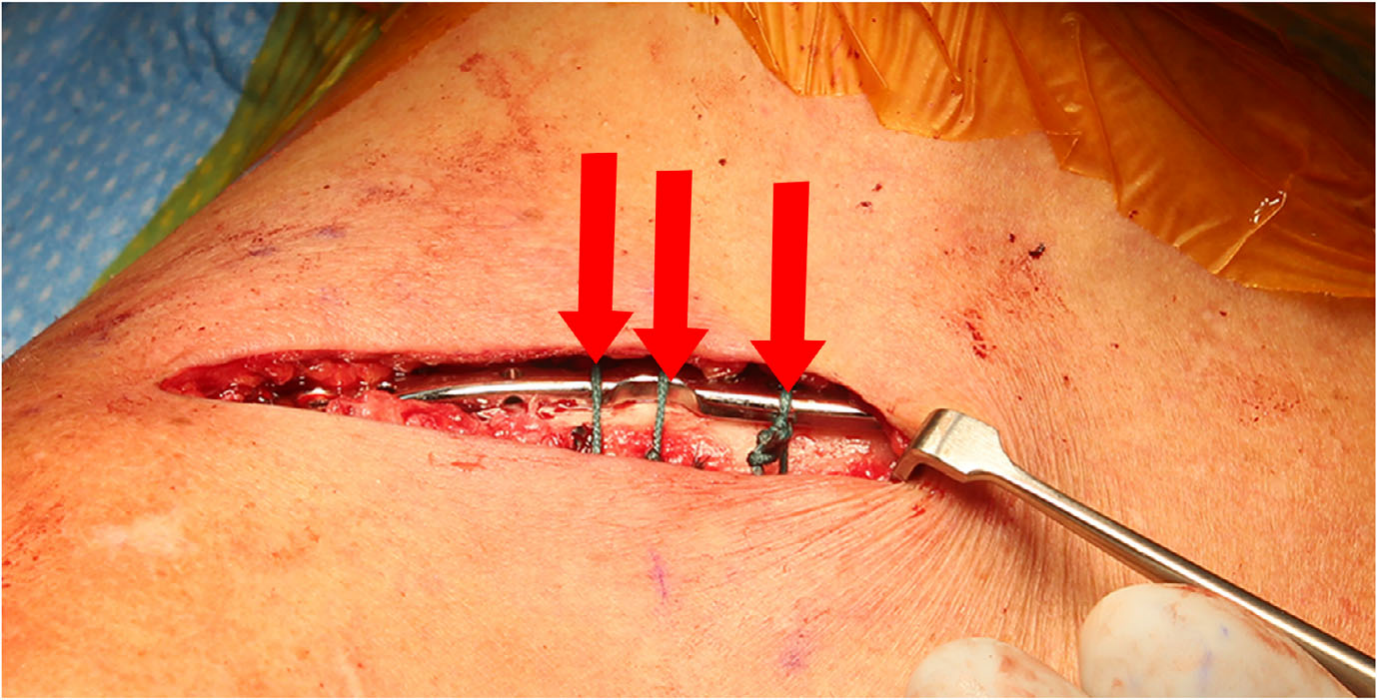
Looking into the superficial incision over the right clavicle fracture. This is the final result of soft cerclage in addition to plate‐and‐screw fixation. Three cerclage loops were used, which are highlighted by red arrows.

Easily accessible superior and anterior fragments are keyed into anatomic reduction and lag screws are employed to promote anatomic osteosynthesis and direct bone healing. Once the remaining fragments have been reduced and alignment is confirmed with intraoperative fluoroscopy, a Synthes 2.7 mm stainless steel variable angle clavicular plate is fitted superiorly to match the contour of the clavicle. Beginning with the proximal and distal ends of the plate, low profile cortical screws are placed for provisional fixation. Additional cortical and locking screws then follow to complete neutralization plate osteosynthesis and to ensure at least 6 cortices of fixation on both sides of the fracture. With comminuted fractures in adults, there is often at least an additional posterior or inferior butterfly fragment that cannot be safely captured using the plate or with conventional lag screw techniques. To address this issue, an Ethibond #5 suture is first passed inferiorly to the clavicle, anterior to posterior, with care to stay directly on bone in order not to entrap and subclavian structures. This is accomplished either by passing the blunt end of the needle or by removing the needle and using a 90° or 30° curved hemostat to facilitate passage. The suture is then retrieved superiorly, tension is obtained manually, and a surgeon's knot is tied and clamped by an assistant to maintain tension. A minimum of 5 additional half hitches is then placed to secure the knot. This process is then repeated as many times as necessary to achieve the desired reduction of posterior and/or inferior fragments (Figure 3). Fluoroscopy, both anteroposterior and axial, is then used to ensure anatomic reduction.

### Closure

Prior to closure, meticulous hemostasis is achieved, followed by copious normal saline irrigation. One gram of vancomycin powder is placed onto the wound for infection prophylaxis. The deep clavipectoral fascia is closed with interrupted 2‐0 Vicryl for watertight closure, followed by 2‐0 Monocryl reapproximation of the platysma, and 2‐0 Monocryl closure at the deep dermal layer. Finally, a running 3‐0 Monocryl is used for the skin, followed by Dermabond and a silver impervious dressing.

### Rehabilitation

**FIGURE 4:**
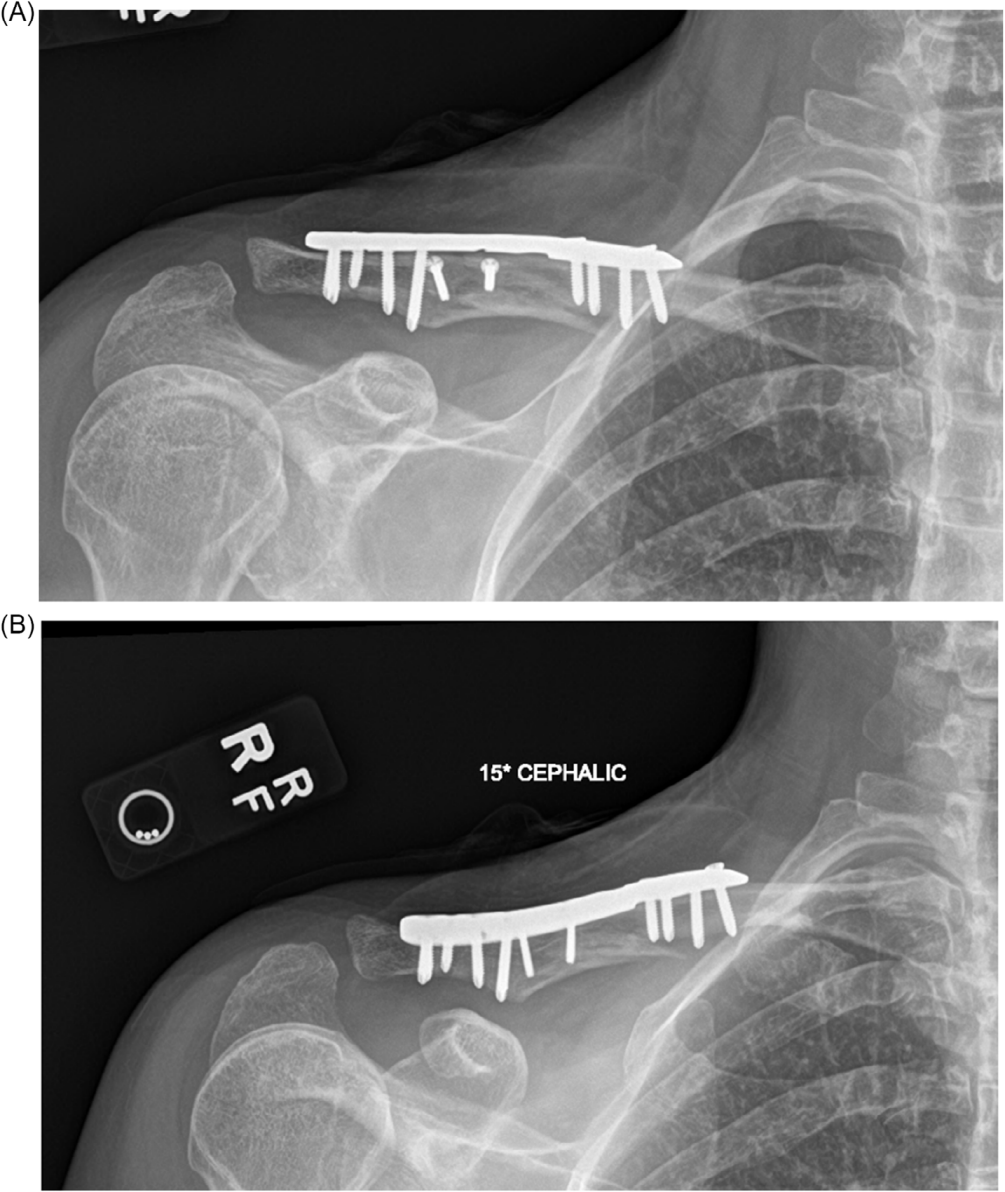
Right postoperative anteroposterior (AP) radiographs with no tilt (A) and with 15° cephalad tilt (B) that were taken 2 weeks status post clavicle ORIF with soft cerclage.

Patients are provided a standard sling to use for 2 weeks postoperatively for comfort and nerve block precautions. Under most circumstances, patients can bear weight as tolerated. Formal physical therapy may begin 1 to 3 days after the procedure. Patients are allowed full shoulder, elbow, wrist, and hand range of motion. Return to sport is expected in 6 to 10 weeks. Postoperative radiographs are obtained at each follow‐up visit to ensure that alignment has remained intact (Figure 4).

## DISCUSSION

Comminuted clavicle fractures are common and management remains controversial.^9^ Because standard plating techniques often fail to capture posterior and inferior butterfly fragments, cerclage is a useful, but uncommonly used option. Typically, cerclage fixation of the clavicle has been avoided because of nearby neurovascular structures in the subclavicular space. With metallic cerclage in particular, wire breakage or migration could pose a significant threat to the brachial plexus and subclavian vasculature.^10^ Nonmetallic cerclage may provide a useful, lower‐risk augmentation technique in many cases.

Based on the authors’ clinical experience, combining suture cerclage with plate‐and‐screw fixation offers a viable and efficient treatment option for displaced, comminuted midshaft fractures without the possibility of hardware prominence. Importantly, prior literature has indicated that cerclage in the humerus and clavicle does not delay time to bony union.^7^, ^11^ Meanwhile, another recent study reported that operative time when using Nice knot suture cerclage was significantly shorter compared with traditional wire techniques.^12^

Disadvantages of suture clavicle cerclage include increased overall operative time and, perhaps, less robust fixation and compression than wire techniques (Table 1). Additionally, risk remains to the neurovascular structures in the subclavicular space, as passage of any cerclage material around the clavicle can injure the subclavian vessels or nerves (Table 2). Nevertheless, this technical note offers a reproducible augmentation technique for superior or anterior plating constructs that is cost effective and safe.

**TABLE 1:** Advantages and Disadvantages of Soft Cerclage in Addition to Plate‐and‐Screw Fixation

Advantages	Disadvantages
Better alignment of inferior and posterior butterfly fragments	Increased operative time and cost
Promotes union of comminuted clavicle fractures	Increased neurovascular risk
Simple, easily implemented technique	Less robust fixation compared with metal cerclage

**TABLE 2:** Pearls and Pitfalls of Soft Cerclage in Addition to Plate‐and‐Screw Fixation

Pearls	Pitfalls
Using the blunt end of the curved needle is a safe way to pass the cerclage suture and should be kept in contact with the clavicle cortex at all times to avoid neurovascular involvement	Suture cerclage does not preclude normal principles of clavicle fracture osteosynthesis, including appropriate plate length to allow for 6 cortices of fixation on each end of the fracture
Once knots are tied in the cerclage suture, the knot stacks should be repositioned either anteriorly or posteriorly but not superiorly to avoid prominence, which can be irritating, especially if positioned superior to the plate	Avoid penetrating inferiorly while passing the suture cerclage, especially medially, to avoid neurovascular compromise
A small, pointed reduction clamp can be used to provisionally reduce the butterfly fragments after suture passage but before tying to anatomically reduce the fragments as much as possible	Avoid excessive dissection of the pectoral and trapezius attachments to the butterfly fragments in order not to devascularize them

## DISCLOSURES

The author (J.J.R.) declares the following financial interests/personal relationships which may be considered as potential competing interests: J.J.R. reports a relationship with Smith & Nephew that includes: consulting or advisory. The other authors (D.A.B., O.M.J., J.H.K., E.K.A.) declare that they have no known competing financial interests or personal relationships that could have appeared to influence the work reported in this paper.
